# Is unassisted smoking cessation choice and success associated with high mental stress? Evidence from six cities in China

**DOI:** 10.18332/tid/193606

**Published:** 2024-10-14

**Authors:** Tingzhong Yang, Randall R. Cottrell, Dan Wu

**Affiliations:** 1 Women’s Hospital, School of Medicine, Zhejiang University, Hangzhou, People's Republic of China; 2 Center for Tobacco Control Research, School of Medicine, Zhejiang University, Hangzhou, People's Republic of China; 3 Research Center for Digital Health Behavior Theory and Management, Zhejiang University National Health Big Data Institute, Hangzhou, People's Republic of China; 4 School of Health and Applied Human Sciences, University of North Carolina, Wilmington, United States; 5 School of Psychology, Shenzhen University, Shenzhen, People's Republic of China; 6 Shenzhen Humanities and Social Sciences Key Research Bases of the Center for Mental Health, Shenzhen University, Shenzhen, People's Republic of China

**Keywords:** unassisted smoking cessation, mental stress, cessation success

## Abstract

**INTRODUCTION:**

Unassisted smoking cessation (USC) is a method of quitting smoking driven by self-determination without the support of professional cessation assistance. This approach may contribute to a potential decrease in overall smoking prevalence within a population. However, the factors potentially influencing smokers’ choice of USC and their success remain unclear. This study examined the associations between mental stress and USC choice and success.

**METHODS:**

Between June and September 2016, a cross-sectional multistage sampling design was used to interview subjects from six selected cities in China. A standardized questionnaire was used to obtain information on sociodemographic characteristics, USC choice, and success. Mental stress was measured by the Chinese version of the Perceived Stress Scale (CPSS). Multivariate logistic regression models were used to examine the association between mental stress, USC choice, and success, with adjustments for relevant covariates.

**RESULTS:**

Among 1647 smokers who had attempted or had quit, 91.6% (95% CI: 90.9–97.5) reported that they had done so without assistance, and 42.1% (95% CI: 32.4–61.3) of them achieved abstinence. While mental stress was not significantly associated with USC success (χ^2^=2.02, p=0.1547), smokers experiencing high levels of mental stress were 0.34 times less likely (95% CI: 0.23–0.50) to attempt USC compared to those with low levels of mental stress. Moreover, a significant negative linear association was observed between varying levels of mental stress and the prevalence of USC use (R^2^=0.910, p<0.001).

**CONCLUSIONS:**

The study findings should help to understand USC and its role in reducing smoking prevalence in the Chinese population. These findings can inform future tobacco control programs and policies in China. Government and social agencies should prioritize understanding smokers’ preferences for USC and providing USC services to promote success within the population.

## INTRODUCTION

Smoking is a leading and preventable cause of illness and death. Quitting smoking reduces the risk of adverse outcomes and benefits long-term health. Many studies found that the success rate of assisted smoking cessation strategies offered by health professionals, including both pharmacotherapies and non-pharmacological methods, is higher than the success rate of those attempting unassisted smoking cessation (USC)^1-3^. However, in the real world, the majority of smokers choose USC methods^4-6^. This is also true in China, where assisted smoking cessation (ASC) methods are admired and widely used by those looking to quit smoking^7,8^. A 2020 study found that 93.1% of those trying to quit selected the ASC method^9^. Other studies showed that 87.6% of those who attempted to quit smoking did so using USC methods, while 97.6% quit without assistance^10^, and 42.1% of smokers who attempted to quit unassisted achieved abstinence. Though the central and local governments established many smoking cessation clinics and hotlines, the number of smokers who use these smoking cessation services is minimal^11^. For example, in 2009, Hangzhou offered 19 smoking cessation clinics, but these clinics had few visitors, with some clinics recording only one visit per month^11^. Similarly, in Beijing, while 22 clinics were established in 1996, only three were retained due to low usage rates^11^. A report shows that the mean annual number of visitors to smoking cessation clinics in China is only 65.67 individuals^12^. In locations such as Hefei city, where a quitting hotline was established in 2015, the results were quite similar to those in Jiangxi province, where hotlines set up earlier on World Smokefree Day in 2009 recorded only nine contacts over six months^12^.

To understand why this situation occurs, a thorough understanding of the nature of USC is necessary. USC consists of two main aspects: the selection of the USC method and the ability to quit successfully. Both aspects can be influenced by individual and environmental factors, including demographic characteristics, level of smoking addiction, individual willpower, and motivation to quit^8,13,14^. Some studies found that factors contributing to the success of USC were financial status, fear of illness, personal health concerns, and family pressure to quit^15-17^. Studies also found environmental factors associated with USC’s use and success, such as tobacco advertising, exposure to anti-tobacco information, and environmental smoking restrictions^8,10^.

According to SCM theory, any stimulus (S) that increases cognition (C) and mental response, can ultimately impact people’s mental and behavioral responses and cause health problems (M)^18,19^. Smoking is considered a way for people to respond to stress. Many studies found mental stress to be associated with smoking^20-22^. Some studies also found mental stress was associated with smoking cessation success^23,24^. Unassisted smoking cessation is quitting smoking through self-determination without relying on professional smoking cessation assistance. However, to our knowledge, there has been no exploration of the association between mental stress and both USC use and success. This study aims to examine these associations.

Generally, it is believed that clinical and other individual smoking cessation methods cannot fundamentally reduce smoking prevalence in the population. However, USC can serve as a population phenomenon that has the potential to decrease the overall smoking prevalence in a population significantly^8^. A study found unassisted quitting has contributed to successful quitting by approximately 70 million smokers in China^9^, which effectively decreased population smoking prevalence and yielded very substantial health benefits. This study aims to identify potential correlates of USC attempts and success, explicitly exploring their association with mental stress. These findings should help in understanding USC and help guide future tobacco control policies and interventions designed to reduce population smoking prevalence.

## METHODS

### Study design, setting, and participants

This study was an observational, cross-sectional, multilevel survey conducted between June and September 2016. It utilized a multistage cluster sampling design. Six cities across China were strategically selected to represent a diverse range of regions based on their geographical distribution: Northeast (Jilin), North Central (Taiyuan), Northwest (Xianyang), Southwest (Chongqing), Central East (Hangzhou), and Southeast (Guangzhou)^10^. Two residential districts were randomly selected from the main urban zones in these cities. Subsequently, four communities within each district and five building blocks within each community were chosen at random. One household was randomly selected out of every twenty from the family household registration list, which was acquired from the building block’s management office. The criteria for selection mandated the inclusion of a male resident aged ≥15 years who had resided in the selected household and lived in any of the six cities under study for a minimum of one year. For the final selection phase, if a household had multiple male residents fitting the criteria, the one whose birthday was closest to the date of contact was chosen for the survey.

### Data collection

After agreeing to participate in the survey, individuals were given a self-administered questionnaire. The surveys were distributed for completion either in the privacy of their homes or in tranquil settings like backyards or community parks. Typically scheduled during weekends, evenings, or other convenient times, these sessions generally took participants about 10 minutes to complete the survey. Privacy was assured as all responses remained anonymous, and participants could request clarifications on any survey question. The ethics committee of Zhejiang University approved the study protocol, and verbal consent was secured from all participants following guidance from an investigator. Upon completing the questionnaire, respondents were compensated with a gift of 10 RMB (about US$1.4, current exchange rate)^8^. This uniform survey protocol was applied across all six cities to ensure consistency in the interviewing process and data collection.

### Measurement

*Dependent variable*

Participants were asked whether they currently smoked. Response options included: ‘Yes, smoke every day’, ‘Yes, smoke on one or more days but not every day’, or ‘No’. Those who answered ‘Yes’ were categorized as current smokers. Successful quitters were defined as people who had a continuous or cumulative smoking history of 6 months or more but were not smoking currently. Smoking intensity consisted of the smoking amount and smoking duration. Quit attempt refers to attempts to quit smoking on at least three occasions (at least three days on each occasion) but not yet successful at the time of this survey^25^. USC refers to the instances when individuals quit smoking without seeking help from healthcare professionals, such as quit clinics, hotlines, or other support services^6,14^.

*Independent variable*

Mental stress was measured by the Chinese version of the Perceived Stress Scale (CPSS)^26^. This scale comprises 14 items that address perceptions of stress during the month before the survey. Items were rated on a 5-point Likert-type scale ranging from 0 (never) to 4 (very often). Item scores were summed to yield a total stress score, with higher scores indicating higher perceived stress levels. This scale has been widely used to assess stress in China and has been shown to have acceptable reliability and validity^26-28^.

*Covariates*

Sociodemographic characteristics included in this study were age, gender, ethnicity, education level, occupation, family location, and household income. Family location was where participants’ families were located at the time of the study, which was categorized into three types: rural area or township, county town or county-level city, and medium or large city. Household income was the average income per person in their households for the previous year.

### Statistical analysis

Data were input into a Microsoft Excel database and subsequently transferred to SAS (version 9.4) for statistical evaluation. We computed descriptive statistics to assess the prevalence of USC use and successful cessation. Using the Rao-Scott chi-squared test, the unadjusted analysis method was built for each primary variable. The multivariate logistic regression models determined associations between mental stress and USC usage or success. Adjusted odds ratios (AORs) and 95% confidence intervals (CI) were calculated. The initial model, the base model, incorporated sociodemographic variables (Model 1). Subsequent models factored in the number of cigarettes smoked and mental stress levels to create the smoking quantity model (Model 2) and the mental stress model (Model 3). For each analysis, we utilized SAS survey procedures, considering the district as the clustering unit, to address within-cluster correlations due to the complexity of the sample. Additionally, we classified the scores of mental stress as different group scales, namely <10, 10–14.99, 15–24.99, 20–24.99, 25–29.99, and ≥30, and explored the linear relationship between mental stress levels and the prevalence of USC use, utilizing scatter plot regression analysis. All statistical tests were conducted using a two-tailed approach, with a significance level of 0.05.

The analyses were weighted to correct for various biases. These weights consisted of: 1) sampling weights, calculated as the inverse probability of selection at both city and district levels and then aggregated; 2) non-response weights, which took into account household and individual factors; 3) post-stratification weights, established based on age groups (<25, 25–34, 35–44, 45–54, and ≥55 years) using demographic distributions from a national survey^29^. The final overall weights were derived by multiplying these three sets of weights together.

## RESULTS

**Figure 1:**
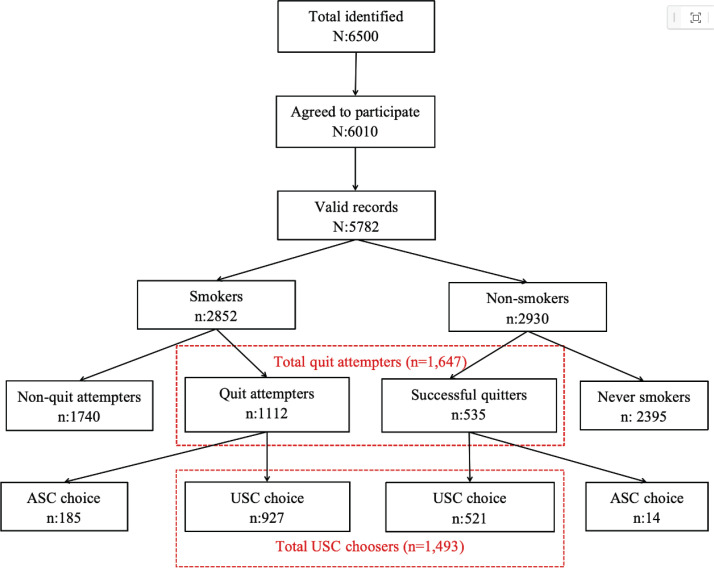
Flow chart for the sample classification, cross-sectional study in six cities of China, 2016 (N=5782)

A total of 6500 individuals were identified as potential participants for this study, of whom 6010 (93.9%) agreed to participate in the survey. Out of the 6010 questionnaires, 5782 valid records were obtained. The sociodemographic characteristics are presented in Table 1. Of the 5782 participants, 2852 were smokers with a prevalence of 44.8% (95% CI: 41.1–48.5). In this sample, 1647 had attempted to or had quit (Figure 1). Of those who had attempted or quit smoking, 91.6% (95% CI: 90.9–97.5) reported doing so without assistance (n=1493). Among current smokers who had attempted to quit, 87.6% (95% CI: 87.6–89.1) reported doing so without assistance, while 97.6% (95% CI: 96.7–98.5) of those who successfully quit did so without help. Of those quit attempters who chose USC methods, 42.1% (95% CI: 32.4–61.3) achieved abstinence.

**Table 1:** Sample characteristics of total quit attempters and prevalence of USC choice (N=1647) and success (N=1493) by weight, a cross-sectional study in six cities of China, 2016

*Characteristics*	*Quit* *attempters* *n*	*Proportion* *of sample* *%*	*Prevalence* *%*	*Rao-Scott* *chi-squared test* *χ^2^ (p)*
**USC choice** (N=1647)				
**Age** (years)				32.08 (<0.001)***
<25	155	9.8	90.9	
25–34	315	16.8	94.7	
35–44	427	19.4	91.6	
45–54	406	23.3	96.0	
≥55	344	30.7	95.2	
**Ethnicity**				4.64 (0.031)*
Han	1566	95.6	94.0	
Minority	81	4.4	97.2	
**Education level**				13.14 (0.011)*
Elementary school or lower	158	17.0	98.8	
Junior high	434	29.9	90.0	
High school	481	23.5	88.3	
Junior college or higher	574	29.5	91.3	
**Marital status**				56.08 (0.009)**
Unmarried	298	18.1	91.4	
Married	1248	75.5	95.0	
Divorced/widowed	101	6.4	89.1	
**Occupation**				17.08 (0.017)*
Managers and service	208	8.7	94.8	
Professionals	140	8.3	94.4	
Commercial and social service	321	18.4	94.7	
Technical workers	492	29.8	94.7	
Operations	188	15.7	93.5	
Retired	59	4.0	92.5	
Students	74	4.9	90.7	
Other	165	10.2	94.7	
**Annual average income per person in household** (RMB)				0.22 (0.974)
<20000	489	30.1	94.6	
20000–39999	504	31.6	94.7	
40000–59999	288	16.2	94.1	
≥60000	366	22.0	92.9	
**Cigarettes per day**				18.77 (<0.001)***
<10	875	56.5	95.4	
10–19	372	18.3	93.1	
≥20	400	25.2	92.6	
**Smoking duration** (years)				5.59 (0.134)
<10	764	51.7	96.1	
10–19	294	14.7	91.5	
20–29	300	14.8	89.4	
≥30	289	18.7	94.8	
**Mental stress**				90.65 (<0.001)***
Low	1050	68.3	94.6	
High	597	31.7	85.0	
**USC success** (N=1493)				
**Mental stress**				2.02 (0.1547)
Low	994	70.5	44.1	
High	499	29.5	37.3	

RMB: 1000 Chinese Renminbi about US$140.

* p<0.05.

** p<0.01.

*** p<0.001.

**Figure 2:**
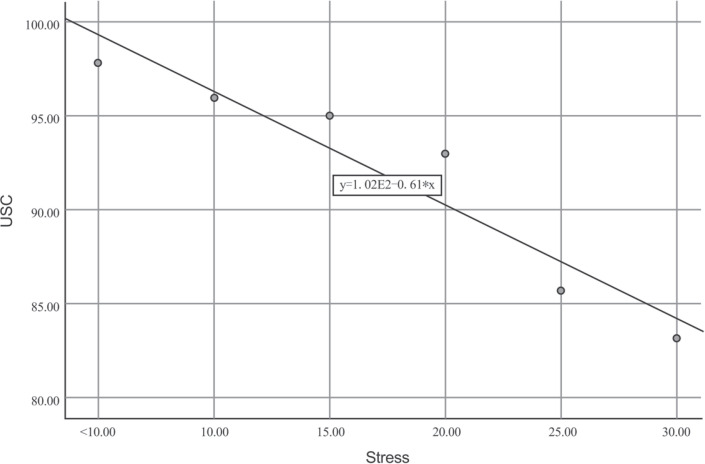
Scatter plot between mental stress score and unaided smoking cessation (USC) prevalence, cross-sectional study in six cities of China, 2016 (N=1647)

Rao-Scott chi-squared test showed that age, education level, marital status, family income, number of cigarettes smoked, and smoking duration were significantly associated with USC adoption (Table 1). Multiple logistic regression analysis revealed that older age was linked to increased use of USC (AOR=2.16; 95% CI: 1.05–4.45) in those aged 45–54 years compared to those aged <25 years. Those in junior high school exhibited lower use of USC than the reference group (AOR=0.57; 95% CI: 0.39–0.85). Number of cigarettes smoked was significantly correlated with lower use of USC (AOR=0.66; 95% CI: 0.44–0.97; for 10–19 cigarettes/day) and (AOR=0.51; 95% CI: 0.41–0.63; for 20 cigarettes/day). High levels of mental stress were found to be significantly associated with lower adoption of USC (AOR=0.34; 95% CI: 0.23–0.50) in the higher mental stress group than the lower higher group (Table 2). Moreover, scatter plot regression showed significant correlations between mental stress score and USC use (R^2^=0.910, p<0.001). The lower the mental stress score, the higher the USC use (Figure 2). The unadjusted analysis found mental stress was not significantly associated with USC success (χ^2^=2.02, p=0.1547) (Table 1).

**Table 2:** Results of multivariate logistic regression analysis by weight, a cross-sectional study in six cities of China, 2016 (N=1647)

*Characteristics*	*Model 1* *(demographic model)* *AOR (95% CI)*	*Model 2* *(smoking quantity model)* *AOR (95% CI)*	*Model 3* *(mental stress model)* *AOR (95% CI)*
**Age** (years)			
<25 ®	1	1	1
25–34	1.43 (0.54–3.77)	1.91 (1.14–3.20)**	2.17 (1.34–3.52)**
35–44	0.89 (0.36–2.21)	1.23 (0.86–1.74)	2.32 (1.05–5.12)**
45–54	2.16 (1.05–4.45)*	2.99 (1.68–5.33)**	3.55 (2.35–5.36)**
≥55	1.66 (0.81–3.43)	2.06 (1.51–2.83)**	3.53 (1.73–7.19)**
**Education level**			
Elementary school or lower ®	1	1	1
Junior high school	0.57 (0.39–0.85)**	0.60 (0.38–0.95)*	0.15 (0.04–0.65)*
High school	1.37 (0.60–3.15)	1.50 (0.55–4.09)	0.16 (0.03–0.75)*
Junior college or higher	1.04 (0.30–3.65)	1.08 (0.27–4.30)	0.21 (0.06–0.73)*
**Cigarettes per day**			
<10 ®		1	1
10–19		0.66 (0.44–0.97)*	0.79 (0.39–1.61)
≥20		0.51 (0.41–0.63)**	0.23 (0.12–0.49)**
**Mental stress**			
Low ®			1
High			0.34 (0.23–0.50)**

AOR: adjusted odds ratio.

* p<0.05.

** p<0.01.

® Reference categories.

## DISCUSSION

This study suggests that 91.6% of quitters reported quitting smoking without assistance, a percentage similar to that found in another study (93.1%) by the Chinese Center for Disease Control and Prevention^9^. It should be noted that USC use prevalence is much higher in China than in the Western world. In Australia, Smith et al.^30^ reported that 54–69% of former smokers reported quitting without formal assistance, while 41–58% of current smokers had tried to quit without assistance. In the United States, previous studies on successful quit attempts have reported unassisted quit rates ranging from 64% to 78%^4,31^. In Canada, Mao and Bottorff ^32^ found that Chinese smokers rarely used cessation aids or services even after they had immigrated to Canada, with only 3 out of 22 participants (13.6%) doing so. Furthermore, the prevalence of success is high (42.1%) in this study, which is also much higher than that in Western society^4,31^. It is important to note that 87.6% of smokers who attempted to quit smoking reported doing so without assistance, while 97.6% of those who successfully quit did so without help^10^. The rate of using USC in the latter is significantly higher than in the former, highlighting the importance of USC for successful smoking cessation. This may be attributed to variations in cultural norms. To a great extent, Chinese culture still adheres to agrarian social mores prioritizing willpower and determination in addressing behavioral challenges^19^. Considering this cultural viewpoint, Chinese agencies should prioritize understanding individuals’ preferences for USC, support their USC decisions, and promote USC success within the population.

As expected, mental stress was negatively associated with USC’s choice. This seems to reinforce studies that have found mental stress to be associated with smoking^20-22^ and cessation^23,24^. SCM argues that any stimulus that increases cognition and mental response can ultimately impact people’s mental and behavioral responses and may cause mental health problems^14,18^. Smoking is often seen as a way for people to cope with mental stressors, so, understandably, mental stress may impact the choice of selecting USC to quit. People with high mental stress may be heavier smokers, and thus, they may be inclined to seek professional help with smoking cessation. In comparison, lighter smokers with lower levels of mental stress may perceive USC as a better choice. USC is quitting smoking through self-determination without relying on professional smoking cessation assistance, which reflects an individual’s willpower and perseverance. From an individual perspective, variables that reflect cognition and determination towards smoking cessation may play a key role in the success of using USC. Mental stress is not inherently linked to cognitive function and determination; therefore, it is not associated with USC’s success. This means that strengthening USC’s success through public education measures might be more effective than focusing resources on mental stress.

This study found increased USC use with age, which also aligns with findings from other studies^5,14^. This may reflect the fact that as individuals age, the prevalence of health problems may increase, leading to a greater need and motivation to quit smoking^25^. It is interesting to note that the number of cigarettes smoked was linked to the choice of unassisted quitting, aligning with findings from other research studies^14,31^. This is because heavy smokers typically have higher nicotine dependence, which can hinder their ability to quit smoking independently, leading to a lower prevalence of USC adoption. Given these findings, government agencies, healthcare organizations, and other relevant stakeholders must address the needs of individuals with high education attainment and those who smoke heavily. These groups may benefit from targeted cessation assistance to improve their chances of quitting attempts and success.

The prevalence of smoking was 44.8% in this study. Among smokers, 57.8% had attempted or had quit, with 91.6% of these participants utilizing the USC method. Among these USC users, 42.1% successfully achieved abstinence. That is, USC contributes to a success rate of 22% for smokers who quit. In 2018, according to a Chinese CDC survey, more than 308 million adults in China were current smokers^33^. Using the USC success prevalence from this study, it can be extrapolated that approximately 68.7 million Chinese smokers could achieve abstinence using USC. Another study examining specific USC numbers found that USC has contributed to the successful quitting of approximately 70 million Chinese smokers^9^. It is worth noting that the figures from both sources are very close, suggesting their reliability. It is indeed a significant number of smokers who have successfully quit. Those who have quit smoking have positively changed their health, lifestyle, and overall well-being.

It is generally believed that clinical and other individual smoking cessation methods may not fundamentally reduce population smoking prevalence. This study offers evidence that USC use can effectively reduce smoking prevalence in the population. However, the USC approach appears to have been overlooked. The World Health Organization (WHO) introduced the six key measures on tobacco control (MPOWER), which are cost-effective and high-impact measures that assist countries in reducing the demand for tobacco. Assisting with tobacco control is one of these measures^34^. Many countries have made significant efforts and achieved considerable results. The Chinese government places great importance on smoking cessation services provided by healthcare professionals, with smoking cessation clinics and hotlines being widely available nationwide. However, the number of smokers who visit these service agencies is minimal^11^. By contrast, most smokers quit without assistance^8,9^. The government and health professional agencies have not addressed the root of the problem regarding this phenomenon. They have been discussing how to strengthen and improve smoking cessation services, but USC has not been given due attention^35^. We strongly recommend changing the current situation, and USC should be included as an essential part of a more comprehensive public cessation strategy while recognizing the continued value of ASC, particularly for certain high-risk populations.

### Policy implications

What is needed is to enhance understanding of USC’s adoption, the significance and role of USC, and actively advocate for the government to prioritize smoking cessation as a critical component of tobacco control. Considering that current services are not well-received by Chinese smokers, the government should restrict investments in and the establishment of cessation clinics and hotlines for the general population unless there is a clinical necessity. The Chinese government and social agencies should prioritize understanding individuals’ preferences for USC, follow their USC decisions, and promote USC’s success within the population. We believe offering professional help to quit tobacco use should include services tailored to USC. For smoking cessation without assistance, utilizing population-based methods such as mass media campaigns on public awareness and community-based initiatives is essential to improve the success rate of USC among the population^35^.

### Strengths and limitations

This study adds valuable insights into USC choice and success among Chinese smokers, drawing on a large-scale nationwide survey conducted with rigorous sampling methods. These findings offer a foundation for informing future tobacco control programs and policies in China. However, several limitations should be acknowledged. Firstly, the cross-sectional design of this study precludes any causal inferences. Secondly, the study’s focus on urban male smokers excludes the experiences of female smokers and rural populations. As such, the findings are not generalizable to the broader population, and gender differences remain unexplored. Future research should include female and rural participants to understand USC across different demographics comprehensively. Thirdly, the study relies on self-reported data regarding smoking cessation, which introduces the potential for recall bias and social desirability bias. Finally, given that the study is situated within Chinese Confucian culture, caution is warranted when extrapolating the results to other cultural settings, particularly those that emphasize instrumental rationality. Future research should consider cross-cultural variations to enhance the applicability of the findings in diverse contexts.

## CONCLUSIONS

In the current study, regardless of whether the quit attempt was successful or not, approximately 90% of cessation attempters opted for unassisted smoking cessation (USC) methods, indicating that USC is a prevalent phenomenon among smokers in China. Notably, more than 40% of those who successfully quit did so without external assistance. This suggests that USC could be a viable component of a comprehensive smoking cessation strategy, potentially enhancing public cessation advocacy efforts. Furthermore, the study identifies mental stress and cigarette consumption as significant factors associated with the choice of USC. Smokers experiencing high levels of mental stress and those with heavier smoking habits were found to be less likely to adopt USC methods, highlighting that these populations may require additional cessation assistance interventions to enhance their likelihood of quitting attempts and success.

## Data Availability

The data supporting this research are available from the authors on reasonable request.
